# Amazing symmetrical clustering in chloroplast genomes

**DOI:** 10.1186/s12859-020-3350-z

**Published:** 2020-03-11

**Authors:** Michael G. Sadovsky, Maria Yu Senashova, Andrew V. Malyshev

**Affiliations:** 1 Institute of computational modelling SB RAS, Akademgorodok, Krasnoyarsk, 660036 Russia; 2 Siberian federal university, Svobodny prosp. 79, Krasnoyarsk, 660041 Russia; 3 V.F.Voino-Yasenetsky Krasnoyarsk State Medical University, P. Zheleznjaka str. 1, Krasnoyarsk, 660022 Russia

**Keywords:** Order, Triplet, Clustering

## Abstract

**Background:**

Previously, a seven-cluster pattern claiming to be a universal one in bacterial genomes has been reported. Keeping in mind the most popular theory of chloroplast origin, we checked whether a similar pattern is observed in chloroplast genomes.

**Results:**

Surprisingly, eight cluster structure has been found, for chloroplasts. The pattern observed for chloroplasts differs rather significantly, from bacterial one, and from that latter observed for cyanobacteria. The structure is provided by clustering of the fragments of equal length isolated within a genome so that each fragment is converted in triplet frequency dictionary with non-overlapping triplets with no gaps in frame tiling. The points in 63-dimensional space were clustered due to elastic map technique. The eight cluster found in chloroplasts comprises the fragments of a genome bearing tRNA genes and exhibiting excessively high *GC*-content, in comparison to the entire genome.

**Conclusion:**

Chloroplasts exhibit very specific symmetry type in distribution of coding and non-coding fragments of a genome in the space of triplet frequencies: this is mirror symmetry. Cyanobacteria may have both mirror symmetry, and the rotational symmetry typical for other bacteria.

## Introduction

Detailed study of a structure of nucleotide sequences is a key issue in up-to-date molecular biology and bioinformatics. Such studies are carried out in two (interconnecting) paradigms: the former is structure-function relationship, and the latter is evolution. A retrieval of the interrelation between structure and function of various biological macromoleculae is a core issue in such studies. Currently, a huge number of publications appears annually on this subject; yet, the problem is still far from any completion. Moreover, some new structures are reported nowadays [1, 2].

Evolutionary value of such studies is rather apparent: comparing various structures found in DNA sequences of various organisms, one expects to retrieve the fine details of evolution ranging from races and species to global ecological systems. Meanwhile, such studies face a number of problems in selection and quality of biological material to be considered. Skipping off possible errors in sequencing and/or annotation of genetic entities, one faces a great complexity of genomes, or even separate chromosomes. Here one has to study a three-sided entity: structure, function, and phylogeny. Quite often all three issues are so tightly interweaved that one fails to distinguish the effects and contributions of each issue separately.

Prokaryotic organisms are rather suitable for this type of researches: bacterial genome is short and consists of a single chromosome. An ambiguity in bacterial taxonomy looks like a pay-off for the genome simplicity of these organisms; the problem in taxonomy grows up, as higher taxa and clades are considered [3, 4]. In such capacity, organella genomes seem to be even better for the studies of the type mentioned above: keeping oneself within the organella of the same type (say, chloroplasts), one avoids any problems related to a diversity of function encoded in a genome. A number of papers aims to study evolutionary processes on the basis of genome sequences peculiarities retrieval [5, 6] or a comparative study of some peculiar fragments of genomes [1, 2, 7–10] of chloroplasts.

Sounding diversity of structures that could be found in DNA sequences is another problem. Surely, the problem hardness depends on the notion of a structure in DNA molecule. Hereafter a structure is stipulated to be a pattern in mutual interlocation of nucleotides manifesting in statistical properties of formally identified short fragments of a genome, i. e. the ensemble of strings of the given length *q*. Moreover, further we shall concentrate on the ensembles of strings of the length *q*=3 (triplets). Henceforth, the list of triplets observed within a genome or its part accompanied with the frequencies of these former is the structure under consideration; see details below.

Indeed, we shall concentrate on the study of mutual location of the points of 63-dimensional space of triplet frequencies, where each point corresponds to a fragment identified within a genome, due to some regular procedure. A cluster structuredness (if any) of those fragments of genomes converted into frequency dictionaries of triplets, in 63-dimensional metric space is the matter of interest. Such approach has been originally explored by Alexander Gorban and co-authors [11, 12], for bacterial genomes. They have found seven-cluster patterns in the fragments distribution, where the specific type of the pattern is strongly ruled by *GC*-content of a genome.

The most popular theory of chloroplast origin, that is the bacterial symbiogenesis theory [13–18], stipulates a relation between some bacteria, and chloroplasts, motivating our study: whether this relation manifests in a similarity of the patterns observed for bacteria [11, 12], and those observed for chloroplasts, or not. Briefly speaking, the answer is negative. Furthermore, chloroplast genomes exhibit rather specific patterns drastically differing them from bacterial genomes.

## Material and methods

178 chloroplast genomes were retrieved from EMBL–bank. We stipulate a genome to be a coherent sequence from four-letter alphabet *ℵ*={A,C,G,T}; the number *N* of nucleotides is the length of the sequence.

### Frequency dictionaries and genome fragmentation

Let fix the length *q* of a window, and the length *t* of a step. Moving the window upright (for certainty) lengthwise the sequence with the step *t* and counting the number *n*_*ω*_ of strings *ω* of the given length *q* identified by window, one gets finite dictionary F_(*q*,*t*)_. Changing the numbers *n*_*ω*_ for frequencies

1 $$ f_{\omega} = \frac{n_{\omega}}{M}\qquad \text{with} \qquad M = \sum_{\omega} n_{\omega}\,,  $$

one gets frequency dictionary *W*_(*q*,*t*)_; *M*=*N* for *W*_(*q*,1)_. Actually, such definition of *W*_(*q*,*t*)_ requires the connection of a sequence into a ring (see details in [11, 12, 19]).

Everywhere further we shall consider the dictionaries *W*_(3,3)_, only. It enlists the triplets counted with neither overlaps, nor gaps between the sequential positions of a window. The choice of *q*=3 and *t*=3 is motivated by apparent biological issues: triplets yield the strongest signal, in DNA sequences, and the step *t*=3 reveals it, since they may correspond to coding positions in DNA sequence.

There could be three different frequency dictionaries *W*_(3,3)_ in dependence on the reading frame position; that latter is called *phase of a fragment* below. Everywhere below we develop the frequency dictionaries $W^{(0)}_{(3,3)}$ for each fragment of a sequence. The *phase* of a fragment was determined to attribute the dictionary: we have changed the reading frame shift of a sliding window for the shift of the starting point of a fragment converted into the dictionary. Whereas a fragment falls into a coding area, then the number of nucleotides was determined from the starting nucleotide of the coding region, and the fragment location. If this distance is divisible by 3, the fragment is assigned with *phase* 0 label; reciprocally, if the residual of the division is equal to 1 (equal to 2, respectively), the fragment is assigned with *phase* 1 (*phase* 2, respectively) label. A fragment fallen into a non-coding area is labeled with *phase junk*. For purposes of our paper, we understand *coding region* widely: we call this way both protein-coding regions and protein-noncoding regions.

To figure out the inner structuredness of a chloroplast genome, we cut it into a set of (overlapping) fragments. To do that, the length of a fragment *L* and the move step *R* alongside a genome have been fixed; we used the figures *L*=603 and *R*=11, in our studies. The motivation for the choice of such figures is following: we need to choose the length *L* of a fragment to be odd and divisible by 3, while the step *R* must be not divisible by 3. Next, the length of a fragment is chosen to be comparable to a gene length. The step length *R* determines the number of points taken into consideration, e. g. for *K*-means clustering; the chosen step figure yields ∼10^4^ fragments (later converted into the points in a metric space). Obviously, both *L* and *R* could be chosen differently, if necessary.

Any frequency dictionary *W*_(3,·)_ maps a sequence into a point in 63-dimensional space. Indeed, the total number of triplets is equal to 64; meanwhile, the linear constraint

2 $$ \sum_{\omega = \mathsf{AAA}}^{\mathsf{TTT}} f_{\omega} = 1  $$

makes remain only 63 ones independent; the frequency of the last one is unambiguously determined from (2). Formally, any triplet may be eliminated; practically, we excluded the triplet exhibiting the least standard deviation determined over the entire ensemble of the fragments.

Apparently, there might be other ways to determine the excluded triplet. For example, it is useful to exclude the variable with maximum value, for some situations; here we followed the described above way, since the least standard deviation of a triplet frequencies observed over a dataset corresponds to the least distinguishability of the objects comprising a dataset, over this variable. Thus, the dimensionality of the space to cluster the frequency dictionaries of triplets becomes equal to 63.

#### The phase of a fragment of sequence

Previously, three types of frequency dictionaries $W^{(0)}_{(3,3)}$, $W^{(1)}_{(3,3)}$ and $W^{(2)}_{(3,3)}$ were discussed. Meanwhile, we developed only one frequency dictionary; that is $W^{(0)}_{(3,3)}$ dictionary. The fragment was then labeled using one of four labels: *phase* 1, *phase* 2, *phase* 3 and *junk*. The label was determined by the location of a fragment within a sequence; to do that, we used the annotation of each genome under consideration.

A fragment was labeled as *junk,* if the fragment contains a half or longer part fulfilled by a non-coding area; *phase* 0, if the center of a fragment falls into a coding region of a genome, and the length of the sequence between the central nucleotide and the start of the coding region is divisible by 3; *phase* 1, if the center of a fragment falls into a coding region of a genome, and the length of the sequence between the central nucleotide and the start of the coding region yields reminder 1 when divided by 3; *phase* 2, if the center of a fragment falls into a coding region of a genome, and the length of the sequence between the central nucleotide and the start of the coding region yields reminder 2 when divided by 3.

For genes (or coding regions) located in the ladder strand, the above mentioned procedure still holds true, but the distance to the central nucleotide of a fragment is determined not from the start position (formally indicated in a file), but from the end of that latter.

### Clustering of frequency dictionaries

As soon as the fragments are converted into the frequency dictionaries $W^{(0)}_{(3,3)}$, each dictionary was labeled with the number of the nucleotide occupying the central position at the corresponding fragment, and with its *phase*. To make the space of frequency dictionaries metric, one must implement a metrics; there is a number of options here (see [20–27] for details). We use Euclidean metrics:

3 $$ \rho\left(W^{[1]}_{(3,3)}, W^{[2]}_{(3,3)}\right) = \sqrt{\sum_{\omega=\mathsf{AAA}}^{\mathsf{TTT}} \left(f^{[1]}_{\omega} - f^{[2]}_{\omega} \right)^{2}}\,.  $$

Here $f^{[j]}_{\omega }$ is the frequency of a triplet *ω* observed in the *j*^th^ frequency dictionary; this index has nothing to do with the frame shift described above.

We studied the distribution of these fragments, in 63-dimensional space using *VidaExpert* software [28]. No special technique for clustering has been used: we identified the clusters *as is*, through visualization. Nonetheless, all the clusters identified through visualization were also identified with *K*-means; thus, those clusters could be verified objectively. In addition, *GC*-content has been determined, both for each fragment, and the genome entirely.

## Results

First, let’s consider the list of chloroplast genomes used in the study, in more detail. The list is quite homogeneous, in terms of the length of sequences; thus, we may not expect any effect resulted from a length difference. Next point is the eliminated triplet choice; the detailed data are provided in [29]. Actually, there are only four triplets eliminated in various genomes: *CGC* (58 entries), *GCG* (113 entries), *GAC* (1 entry) and *TAA* (also 1 entry).

The triplets *CGC* and *GCG* are of great interest: they both are palindromes (read equally in opposite directions), and besides they together comprise the couple of so called *complementary palindrome*. That latter consists of two strings (triplets, in our case) read equally in opposite directions, with respect to Chargaff’s substitution rule: *CGC*⇔*GCG*. Such symmetry is rather important both in analysis, and in biological issues standing behind it; more detailed discussion see below.

### Eight cluster structure of chloroplast genomes

Let now consider the patterns of chloroplast genomes. To do that, we just located the points corresponding to frequency dictionaries of the fragments of a chloroplast genome, in 63-dimensional space. The best way to see a pattern provided by distribution of the fragments converted into frequency dictionaries is to see it in the space determined by three principal components [30].

To begin with, we shall expand the labeling system described above. Previously, four labels have been introduced: *phase* 0, *phase* 1, *phase* 2 and *junk*. Now we add one more phase called *tail*, and split each *phase* *j*^th^ into two subphases: these are the phases *F*_0_, *F*_1_, *F*_2_, and *B*_0_, *B*_1_, *B*_2_, respectively. The sense of these subphases is clear and apparent: they correspond to forward reading (*F*_0_, *F*_1_ and *F*_2_) and backward reading (*B*_0_, *B*_1_ and *B*_2_) of genes, in leader and ladder strand, respectively. The index coincides to the reminder of the division of the distance between the start position of a coding regions, and the center of a fragment, by 3.

The *tail* phase looks most intriguing. It comprises the fragments falling into a dense series of tRNA (5S RNA, 25S RNA, etc.) genes. We used standard PCA to visualize the data. Consider several genomes shown in two projection: the former is in the plane provided by (*PC*_1_,*PC*_2_) and the latter is in the plane provided by (*PC*_2_,*PC*_3_).

**Fig. 1:**
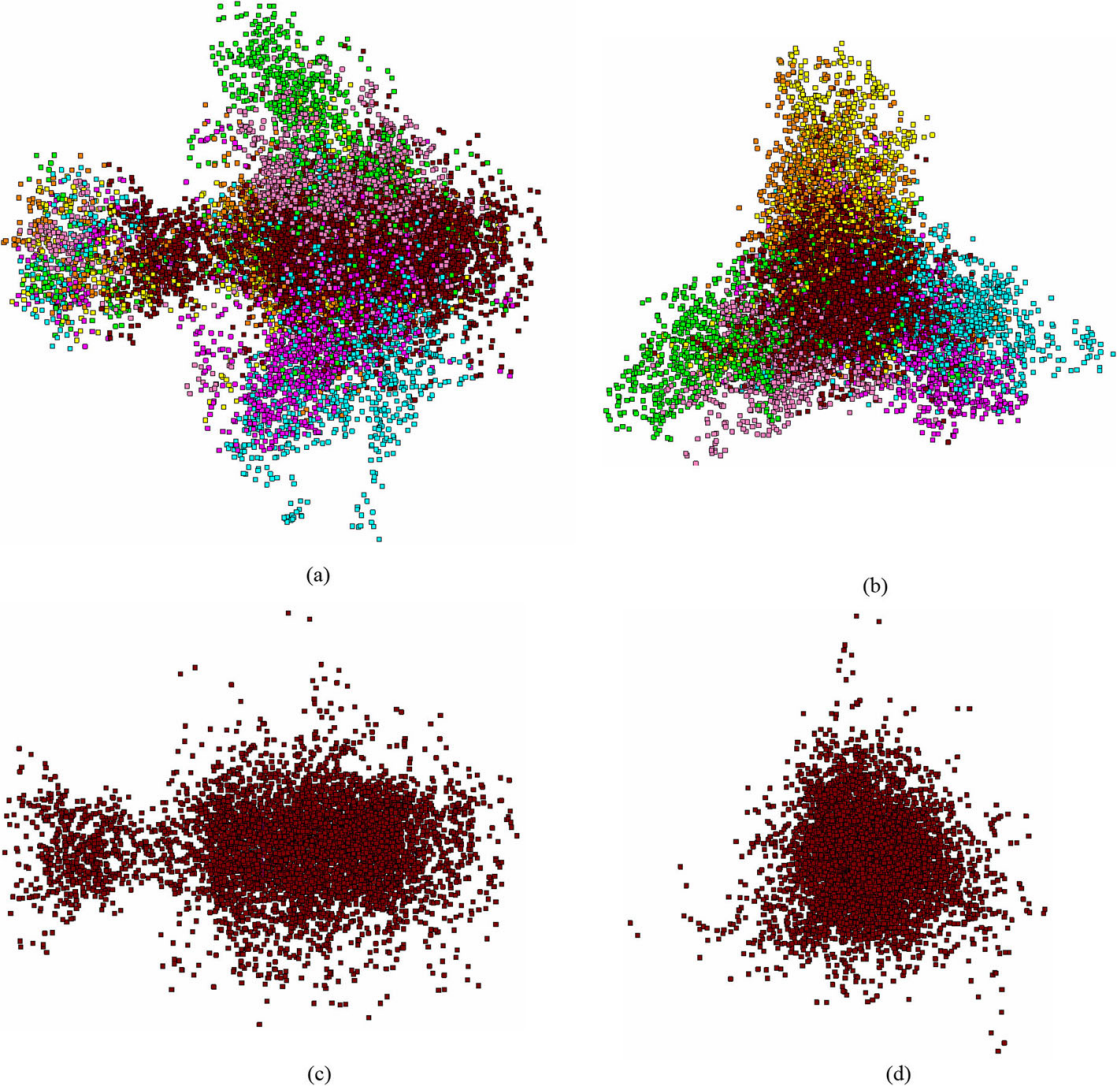
The distribution of 12 244 fragments of *Lolium perenne* chloroplast genome (AC AM777385), in principle coordinates. Subfigures (**a**) and (**b**) show the general overview, subfigures (**c**) and (**d**) show the junk fragments, only. Subfigures (**a**) and (**c**) show the view in (*PC*_1_,*PC*_2_) plane, and subfigures (**b**) and (**d**) show the view in (*PC*_2_,*PC*_3_) plane. The phases are colored as following: *F*_0_ is raspberry box, *F*_1_ is cyan box, *F*_2_ is yellow box, *B*_0_ is rosy box, *B*_1_ is green box and *B*_2_ is orange box

Figure 1 presents (*PC*_1_,*PC*_2_) view of the fragments distribution of ray grass (*Lolium perenne*, AC AM777385 in EMBL–bank) genome, the coding regions. This is a typical “bullet-like” pattern of the distribution (Fig. 1a and c). Figure 1b shows a standard (for chloroplasts) three-array pattern.

Thus, eight clusters are distinctively identified, in this Figure: six ones correspond to six phases (from *F*_0_ to *B*_2_, respectively), the seventh cluster comprises the *junk* labeled fragments, and the eighth cluster (that is the *tail*, see Fig. 1a and c, in the left). Actually, six clusters corresponding to six phases as they are shown in Fig. 1 are located pretty close each other pairwise: *F*_0_−*B*_1_, *F*_1_−*B*_0_ and *F*_2_−*B*_2_. Careful examination of Fig. 1 shows that the fragments (converted into *W*_(3,3)_ frequency dictionaries) gather into dense and clearly identified groups; coloring of the fragments according to their phase unambiguously present these clusters. Simultaneously, it is evident that some phase identified cluster coincide or merge into a common one. For example, it is always so for the cluster comprising the *F*_2_−*B*_2_ phase fragments. Later, it will be shown that this is not so for other genomes (cyanbacterial, bacterial). In such capacity, a formal implementation of some custom clustering methods (e. g., *K*-means) may fail to distinguish the subclusters belonging to different phases observed within a common cluster. For chloroplast genomes, the cluster comprising *F*_2_−*B*_2_ fragments may never be split into subclusters; two other clusters (also mentioned as arrays) may differ in the form, for various genomes. For some genomes, *F*_0_−*B*_1_ and *F*_1_−*B*_0_ clusters are looking as an entity; other genomes exhibit the patterns where these two clusters are split into two arrays each (see Fig. 1b), and these arrays observed within a cluster may be detected by some clustering technique.

Another feature of this genome is the clearly visible *tail* phase, in the distribution of fragments. This is very frequent pattern observed among the studied genomes. The difference between the dictionaries $W^{(0)}_{(3,3)}$, $W^{(1)}_{(3,3)}$ and $W^{(2)}_{(3,3)}$ manifests in the clustering in “wings” (shown in color in Fig. 1); on the contrary, the lack of such difference observed for *junk* phase fragments results in a ball-shaped distribution of these points, in 63-dimensional space. Figures 1c and d show the *junk* phase fragments, solely. Unlike the bacterial genomes [11, 12], here *junk* exhibits the separation into two subclusters (see Fig. 1c).

**Fig. 2:**
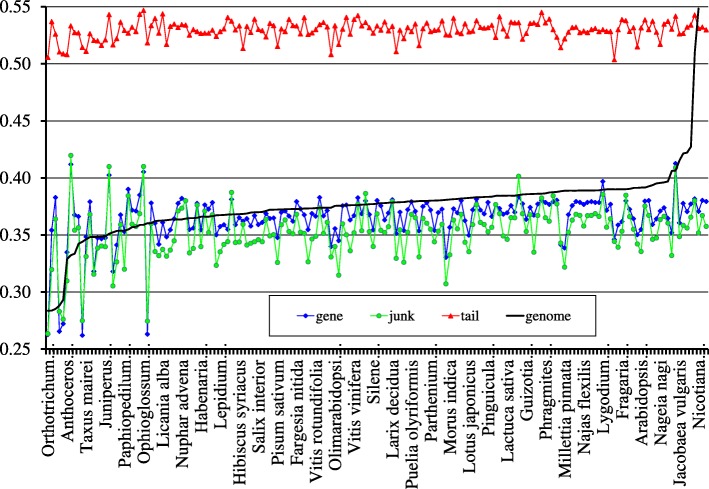
Average *GC*-content for entire genome, coding, non-coding parts and *tail* phase

**Fig. 3:**
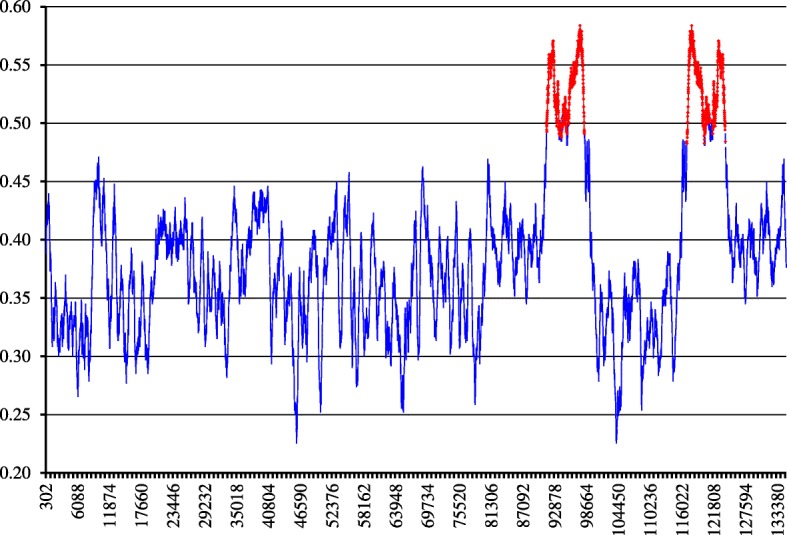
The plot of *GC*-content of all the fragments layered out alongside the *Lolium perenne* chloroplast genome; the *tail* phase (Fig. 1) is shown in red

Let now consider the fragments comprising the tail in more detail. To do that, we determine *GC*-content both for the entire genome, and for each fragment, and plot then the content against the number of a fragment. Figure 3 shows this plot; the *tail* phase is colored in red. Let us remind, that the genome-wide *GC*-content of this entity is equal to 0.38. The overall *GC*-content has been reported to be the key factor defining the structure of clustering of the fragments formally identified within a bacterial genome [11, 12]; that former has significantly less effect, for chloroplast genomes. For chloroplast genomes under consideration, *GC*-content varies from 0.28 (*Orthotrichum rogeri*, AC KP119739 and *Syntrichia ruralis*, AC FJ546412) to 0.55 for *Selaginella uncinata*, AC AB197035. Figure 2 shows more detail data.

This Figure shows the set of chloroplast genomes under consideration ordered with respect to the genome-wide *GC*-content value. Besides, this Figure shows the plots of average *GC*-content determined over the ensemble of coding fragments (all six phases), of average *GC*-content of non-coding fragments, and of average *GC*-content of *tail* phase fragments. Evidently, the plots of genome-wide, coding and non-coding *GC*-content figures exhibit a high concordance in behaviour, while the *tail* phase fragments ensemble remains rather permanent.

Table 1 shows the correlation coefficients determined between averaged figures of *GC*-content of four ensembles of the fragments of genomes. The figures shown in Table 1 reveal the relative independence of the *tail* phase from the other parts of a genome, and *GC*-content of that latter never falls beyond 0.50 level. Greater part of the genomes yield *GC*-content value ranging from 0.35 to 0.40. The set of genomes with lower values of *GC*-content comprises the species *Orthotrichum rogeri*, *Syntrichia ruralis*, *Physcomitrella patens*, *Marchantia polymorpha*, *Sanionia uncinata*, *Anthoceros angustus*, *Ptilidiumpul cherrimum*, *Equisetum arvense*, *Glycyrrhiza glabra*, *Trifolium subterraneum*, *Orobanche gracilis*, *Taxus mairei*, *Millettia pinnata*, *Pisum sativum*, *Juniperus virginiana* and *Juniperus bermudiana*. The genomes of *Aneura mirabilis*, *Lygodium japonicum*, *Pteridium aquilinum*, *Ophioglossum californicum*, *Marsilea crenata* and *Myriopteris lindheimeri* comprise the opposite group with higher figure of *GC*-content. Finally, two species (these are *Selaginella moellendorffii* and *S. uncinata*) yield the highest level of *GC*-content.

**Table 1:** Correlations between *GC*-content of whole genome, coding areas and *junk*

	Gene	junk	*tail*
Genome	0.9745	0.9617	0.6248
Gene		0.9218	0,6285
junk			0.5939

Let now focus on the behaviour of the *GC*-content of the fragments comprising *tails* in the distribution of the fragments (see Fig. 2). Remarkably, there is no genome with *GC*-content figure lower than 0.5, for these fragments. Differing in this figure from the entire genome, the *tails* ensemble still comprises both coding, and non-coding fragments. The former are presented by densely located tRNA genes, 5S RNA and 16S RNA genes. This fact holds true for all genomes exhibiting a *tail* phase, and such genomes make a majority of entities studied in this paper.

Let now provide some examples of the fragments distributions observed in chloroplast genomes with various *GC*-content values. Consider the moss *Physcomitrella patens* (AC AP005672) genome with *GC*-content equal to 0.29 (next to the lowest one in the list of studied genomes).

**Fig. 4:**
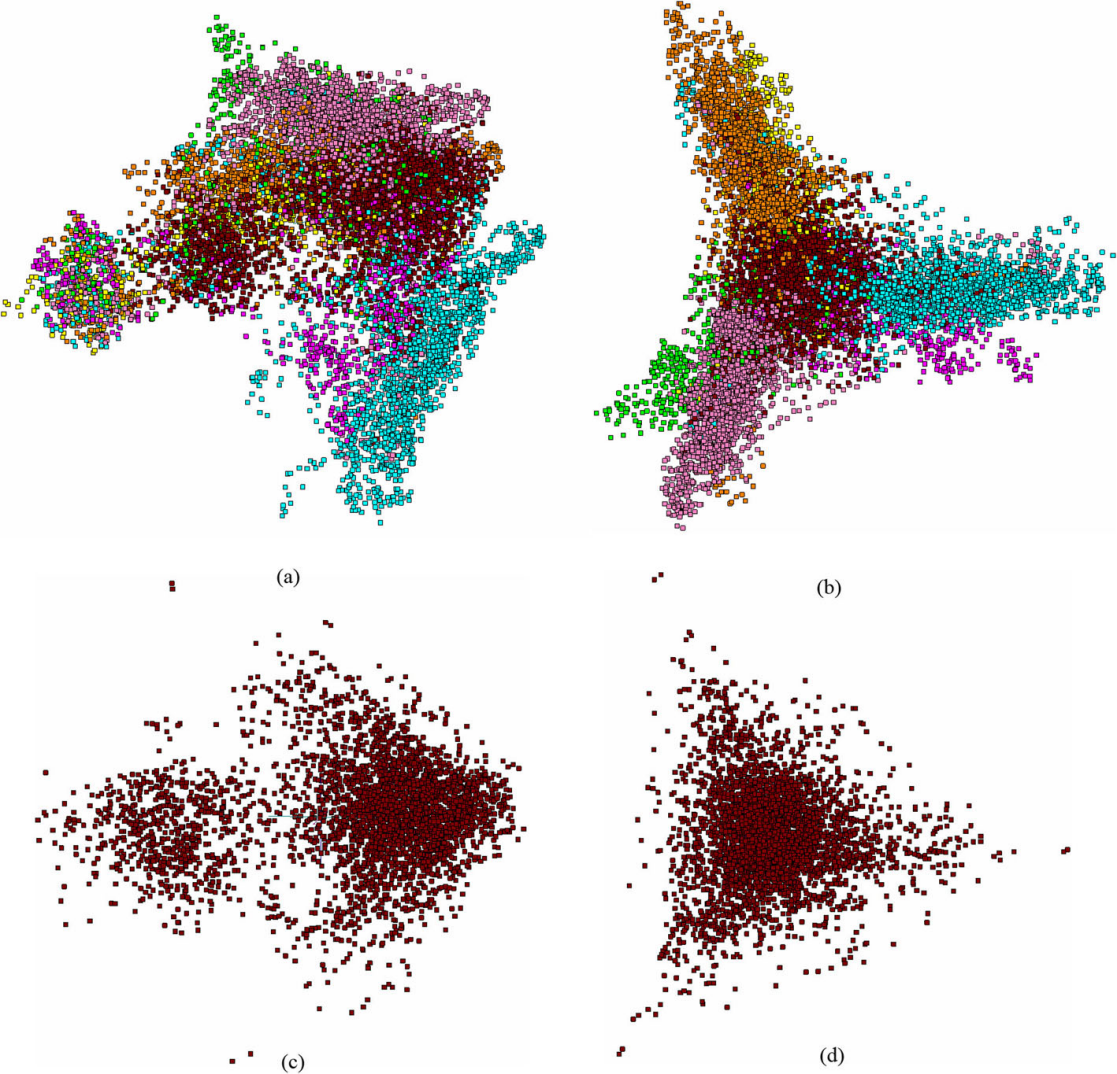
The distribution of 11 118 fragments of moss *Physcomitrella patens* chloroplast genome (AC AP005672). (**a**) and (**c**) are (*PC*_1_,*PC*_2_) view, (**b**) and (**d**) are (*PC*_2_,*PC*_3_) view; (**a**) and (**b**) shows the distribution of all fragments, (**c**) and (**d**) show the distribution with coding fragments erased. Coloring and placing is the same as in Fig. 1

This is the model organism often used in evolutionary studies. Figure 4 shows two projections of the full plot of the fragments; one easily can see similar pattern with two “tripods” overlapping each other, and the *tail* phase part. It should be stressed, that this genome exhibits another triplet with the least standard deviation figure: *CGC*, on the contrary to that one shown in Fig. 1. This genome exhibits stronger split of two phases (these are *F*_1_ vs. *B*_0_ and *F*_0_ vs. *B*_1_), in comparison to the pattern shown in Fig. 1.

To make it more clear, we show the distribution of all the fragments (the color labeling is the same as in Fig. 4) falling in coding regions, only; all the points corresponding to *junk* phase are erased. Figure 4c and d show the distribution of *junk* phase fragments of the moss genome. Similar to Fig. 1c and d, this genome also exhibits an occurrence of some points of *junk* in *tail* phase. Again, it should be kept in mind, that all the distributions shown in Fig. 4 are not independent: actually, all these figures just show the same distribution, while some points are not shown in various figures; still, they affect the distribution pattern.

**Fig. 5:**
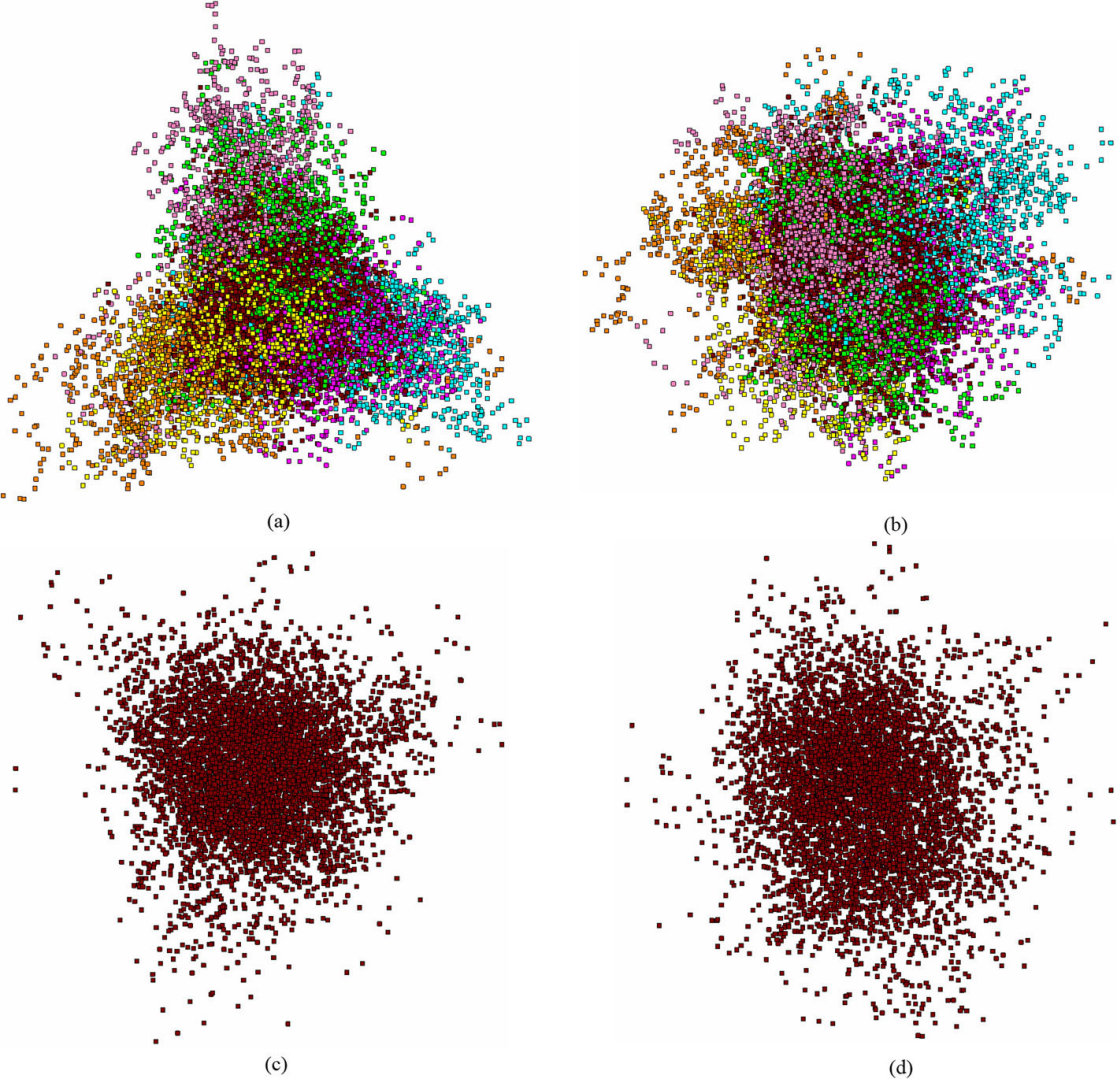
*Selaginella moellendorffii* chloroplast genome fragments distribution; (**a**) and (**b**) show the distribution of all the fragments, (**c**) and (**d**) show the distribution of non-coding fragments (coding ones are erased); (**a**) and (**c**) are (*PC*_1_,*PC*_2_) view, (**b**) and (**d**) are (*PC*_2_,*PC*_3_) view

The patterns shown in Figs. 1, 2, 3, 4 and 5 present a typical structuredness in a distribution of the small fragments of chloroplast genome. Actually, all the genomes except two entities exhibit such pattern in fragments distribution; these latter are the genomes of *Selaginella moellendorffii* (AC HM173080) and *S.uncinata* (AC AB197035). They are extremely ancient and rather isolated mosses belonging to primitive vascular plants. First of all, they have other triplets with the least standard deviation: *GAC* and *TAA*, respectively. Figure 5 shows the distribution of all phases of *S.moellendorffii* genome. There is no *tail* phase at all, in this genome, neither in coding phases, nor in non-coding one. The pattern of distribution for *S.uncinata* is pretty close to that one shown in Fig. 5. Another indirect evidence for this issue is discussed in [16] (see also very useful paper [31]).

### Chloroplasts and cyanobacteria

The difference in the structuredness of a genome of chloroplast from bacterial genome is the key issue of the work. Still, the question arises whether this difference is essential. In other words, while chloroplasts form a tight and uniform group of genome bearers, bacteria are extremely diverse, both in genetics, phylogeny, taxonomy, physiology and ecology. What if there are some bacteria that had fallen out from our analysis, but still are close to chloroplasts, in the sense of the small fragments distribution? Indeed, the diversity of bacteria is huge, and there is no guarantee of the total absence of the coincidence of the structure described above when retrieved from some bacterial genome.

**Fig. 6:**
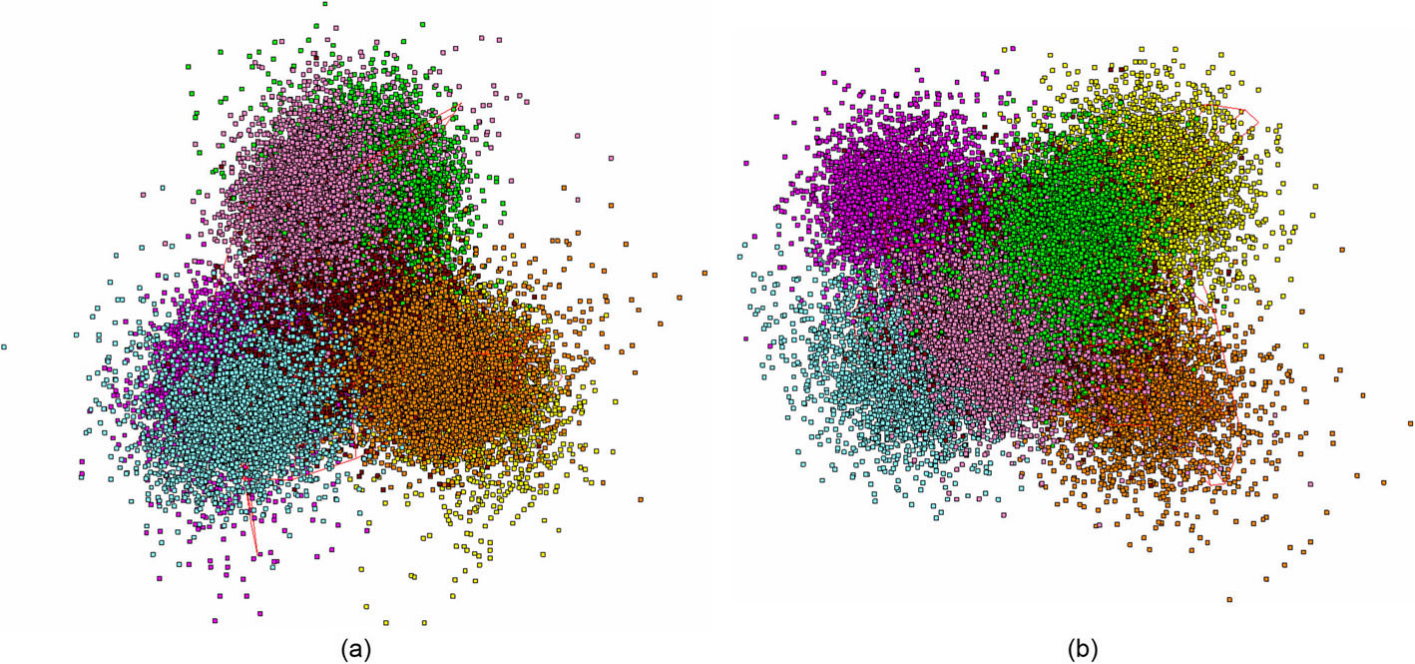
*Nostoc sp.* PCC 7107 distribution of fragments, *Δ*=1005, *R*=202. **a** (PC _1_; PC _2_). **b** (PC _2_; PC _3_)

Speaking on the similitude or any other semblance of the patterns observed in chloroplast genomes to those observed in bacterial genomes, one should first of all concentrate on the comparison of the structures of chloroplasts, and cyanobacteria. These latter are stipulated to be the other branch of descendants of the common ancestor of chloroplasts and modern bacteria. Here we do not study this point in detail, while some preliminary results [32] show that the divergence between chloroplasts and cyanobacteria is tremendous. Figure 6 illustrate the point.

**Fig. 7:**
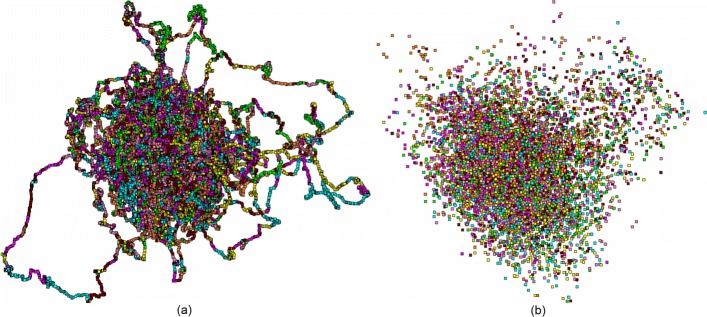
*Gloeocapsa sp.* PCC 7428 (left) and *E.coli* K-12 (right) distribution of fragments, *Δ*=30003, *R*=601. **a** (PC _1_; PC _2_). **b** (PC _1_; PC _2_)

Meanwhile, our observation (not presented here in detail) shows that cyanobacteria differ rather strongly from chloroplasts and the difference seems to be systematic. As a rule, the fragments corresponding to different phases do not gather into a common cluster; thus, the distribution pattern observed for cyanobacteria is pretty close to that one observed for other bacteria (seven-cluster mode). Nonetheless, few cyanobacteria exhibit the pattern resembling that one observed for chloroplasts: the points corresponding to two different phases comprise the same cluster. Unlike for chloroplasts, there is no regularity in the phase occurrence in such “joint” cluster: these might be *F*_2_−*B*_2_ points, as well as other combinations (say, *F*_0_−*B*_1_, etc.). Simultaneously, there is one more feature differing cyanobacteria from other bacteria: a growth of fragment length to *L*=30003 and the step to *R*=601 results in appearance of clearly identified loops provided by the series of the fragments, and these loops are peculiar for cyanobacteria only; there are no such loops in other bacteria, see Fig. 7.

## Discussion

Let now get back to the labeling system (see page 9) of the formally identified fragments in a sequence. It provides a reasonable balance between the impact of coding and non-coding regions. Since the label value depends on the central nucleotide position, then approximately a half of the “border” fragments (i. e. those that cover the border between coding and non-coding regions in a genome) are labeled as *junk*, and another half are labeled as coding ones, with the specific phase value. Suppose, the total number of coding regions in a chloroplast genome is 50. Then an approximate number of “border” fragments labeled as *junk* is estimated as

4 $$ \frac{L}{2R}\times 50 \times 2 \approx 2\,500\,,  $$

where the factor 2 counts both forward and backward oriented coding regions. The same number (4) of the “border” fragments would be labeled with some phase figures; this parity guarantees, to some extent, a lack of distortion in the fragment clustering.

Papers [11, 12] present an approach to figure out a structuredness in bacterial genomes based on systemic and sequential comparison of frequency dictionaries of the fragments of a genome; the fragments were identified in the same way, as we have done. It should be stressed that such fragments were identified regardless a functional charge of a fragment. The results presented in these papers show that the fragments belonging to the same strand tend to cluster arranged in the vertices of two triangles (one for leading strand, and other for ladder one). The triangle vertices comprise fragments of the same phase. A mutual placement of these two triangles is completely determined by the average (over the genome) value of *GC*-content, for bacterial genomes.

A general seven-cluster structure was reported, for bacterial genomes, in these papers; the seventh cluster comprises the fragments falling into a junk area of the genome. The papers [11, 12] also provide an elegant explanation of an origin of this seven-cluster structuredness, describing the constraints forcing two triangles to rotate and project one over another. Here the genome-wide *GC*-content is claimed to be the only key factor determining the pattern of the cluster structure. A minor variation of *GC*-content results in visible change of the structure pattern.

There are following patterns of the fragments distribution, observed on bacterial genomes, for various figures of *GC*-content. *GC*-content close to 25 % yields two “parallel triangles” (for *AT*-reach genomes); the growth of *GC*-content to ∼35 *%* yields the pattern with two “orthogonal triangles”, and the raise of *GC*-content up to 60 % results in degeneration of two triangles into a single plane. Besides, the authors of [11, 12] claim such seven-cluster pattern be universal one; meanwhile, our results disprove this hypothesis.

### Cluster structure of chloroplast genomes

Since chloroplasts take their origin from bacteria [13, 14, 17, 18], then one may expect they inherit this universal pattern of the inner genome structuredness. Our results disprove this assumption; moreover, *GC*-content of chloroplast genomes does not impact the pattern of fragments distribution. The newly found pattern in small fragments distribution in 63-dimensional triplet frequency space seems to be universal: there are two only exclusions from the list of studied genomes [29]. They are presented by two ancient moss species (*Selaginella moellendorffii* and *Selaginella uncinata*) originated more than 4×10^8^ years ago.

**Fig. 8:**
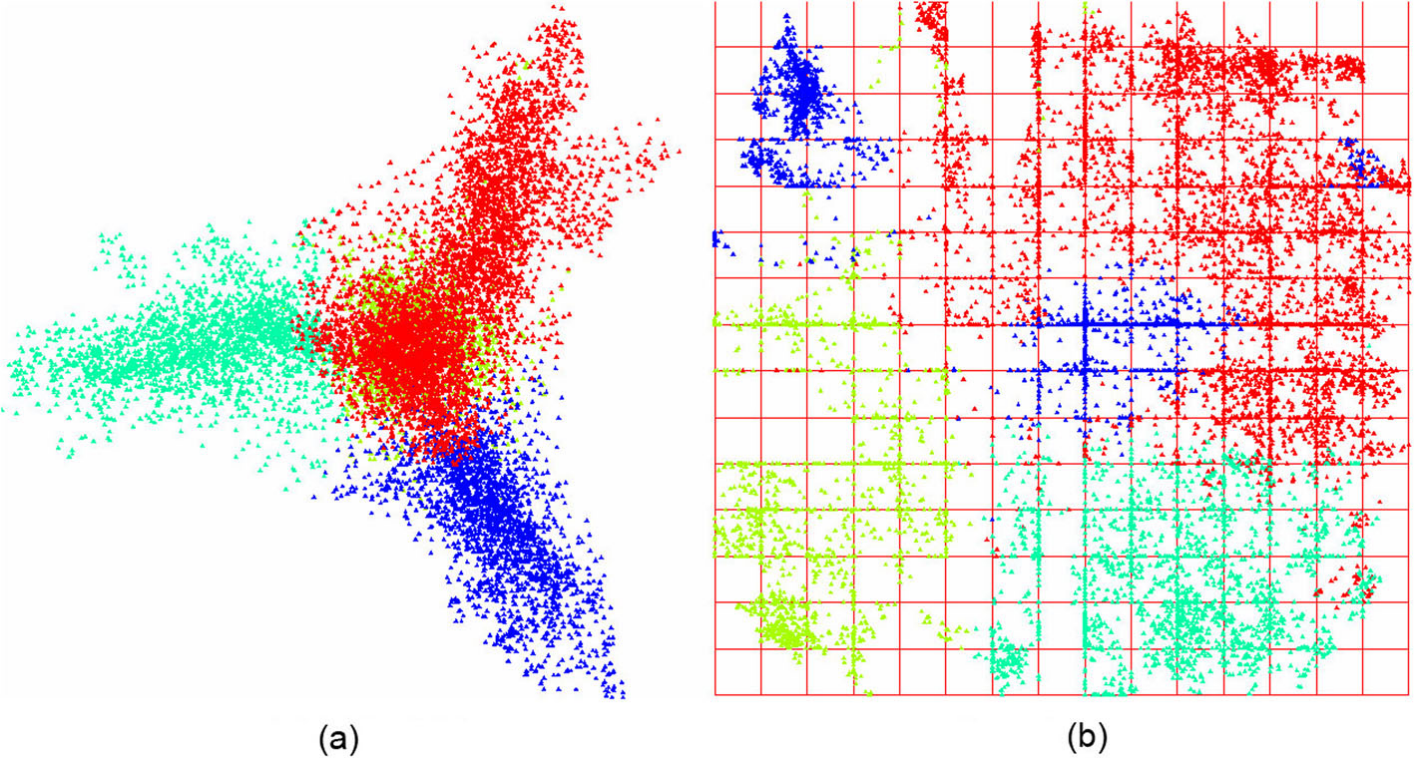
*K*-means (*K*=4) for *Physcomitrella patens* (AC AP005672) chloroplast genome. **a** (PC _1_; PC _2_). **b** inner coordinates

Another important question here is whether the observed groups of points (the phases are colored in figures) corresponding to six phases (these are *F*_0_, *F*_1_, *F*_2_, *B*_0_, *B*_1_ and *B*_2_) really comprise clusters, or it is a kind of artifact resulted from a visualization technique. This question has obvious and transparent answer: yes, the clusters observed by visualization of the phases are the real clusters identified with a clustering technique. To check it, we have carried out *K*-means cluster implementation, of the frequency dictionaries corresponding to the fragments. Figure 8 shows the clustering developed by *K*-means [30] (with *K*=4) for the moss genome. Again, we did not aim to figure out some cluster structure due to *K*-means, but to verify the cluster structure observed in genomes through the visualization (that is the phase coloring). The clustering shown in Fig. 8 is stable: the greatest majority of the points corresponding to different fragments occupy the same cluster. Of course, some points change their class attribution, but the number of such volatile points was small enough. That is true for the points corresponding to coding fragments.

The fragments comprising *tail* part of the distribution always make a separate cluster. It should be stressed that the non-coding fragments (*junk*) merge into a cluster comprising the coding fragments, in different *K*-means runs. There is no obvious regularity in the class merging: the non-coding fragments may join any of three clusters comprising the coding fragments.

Few words should be said towards the pictures shown in Fig. 8. The left picture presents the distribution of all the fragments (of course, converted into frequency dictionaries) in 63-dimensional space, in principal components, the (*PC*_2_,*PC*_3_) projection and clustered into four classes by *K*-means. Obviously, the classes identified by *K*-means comprise the points belonging both to some coding phase, and to the non-coding phase; yet, we did not aim to separate the points by *K*-means in the same manner, as by coloring. The right picture shows the same distribution in so called inner coordinates of an elastic map; the details on this techniques could be found in [22–27].

Careful examination of Figs. 1, 4 and 5 shows the general situation in localization of the phases, within a pattern. Indeed, the localization of the phases could be described by the following cyclic diagrams: *F*_0_→*F*_1_→*F*_2_→*F*_0_ (clockwise), and *B*_0_→*B*_1_→*B*_2_→*B*_0_ (counterclockwise). In fact, these two diagrams mirror each other, so that no complete coincidence might take place due to rotation. Such mirror symmetry corresponds to the double-stranded structure of DNA; the localization of *F*_2_ and *B*_2_ phases in the same projection is here of greater importance. All the studied chloroplast genomes exhibit such mirroring symmetry, while there are no evidences for that latter in bacterial ones [11, 12]. The phases *F*_0_, *F*_1_, *F*_2_ make a triangle with given vertices circuit direction; same is true for the phases *B*_0_, *B*_1_ and *B*_2_, and the circuit direction is the same, as for *F*-phases. This fact seems to be universal for bacteria (and some other genomes, e. g. fungi ones); on the contrary, chloroplast genomes exhibit exactly opposite pattern: they have counter-directed circuit directions, for those phases. Papers [16, 33] report on another type of structuredness found in chloroplast genomes, while we believe the mechanism staying behind these structures and those we are showing here, is the same: triplet frequency peculiarities. More specific mechanism based on codon bias yields a structuredness reported in [34]. These facts may reveal the “independent” evolution of chloroplast genomes (see also [15, 35]), on the contrary to the synchronized evolution of these latter with the host nuclear genome [19]. Also, such symmetry may answer the question towards the attribution of contigs for de novo assembling genomes [36–38] (see also another sight on the problem in [39]).

**Fig. 9:**
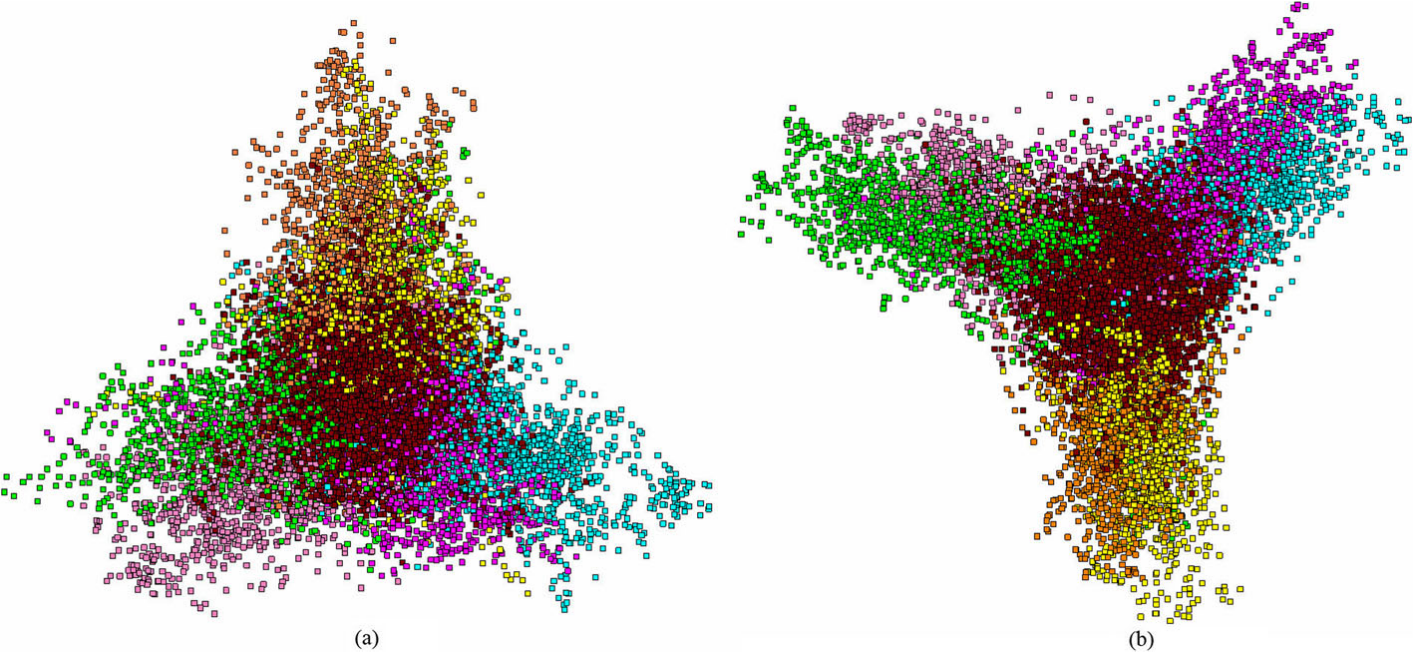
“Up” and “down” orientation of the clusters shown on two genomes: *Ranunculus macranthus* (left), and *Anthoceros angustus* (right). **a** “up”. **b** “down”

This mirroring has one more manifest in mutual location of the clusters comprising different phases. Figure 9 illustrates this fact: while the location of phase 0 and phase 1 remains the same, in both subfigures, the location of the phase 2 takes mirroring positions. The phase 2 cluster faces down, for *Anthoceros angustus*, and that former faces up for large buttercup (*Ranunculus macranthus*). To make the images more apparent, we have erased the points corresponding to junk. Two positions of phase 2 cluster correspond to two mirroring axes systems. Comparing Figs. 1, 4, and 5 (see “Chloroplasts and cyanobacteria” subsection), one sees that such mirroring symmetry is universal, for chloroplast genomes; cyanobacteria that are claimed to be evolutionary related to chloroplasts, do not exhibit such pattern, at all.

Another sounding manifestation of the symmetry is the interchange of the triplet yielding the least standard deviation figure; see again [29]. Indeed, with exclusion of two triplets (these are *GAC* and *TAA*), all other entries exhibit either *GCG*, or *CGC* triplet yielding the least standard deviation figure. The unconventionality of the triplets yielding the least standard deviation figure may result from this long isolated lineage.

Apart these two species, all other ones could be split into two groups: the former with *GCG* triplet yielding the least standard deviation, and the latter with *CGC* triplet; the abundances of each groups are 115 and 61 entries, respectively. It should be mentioned that two genomes were not annotated, completely; thus, we were not able to determine what type of symmetry they exhibit. Table 2 summarizes the distribution of chloroplast genomes over the combinations of U⇔D variants, and the triplets *CGC*⇔*GCG*. Here the label U (D, respectively) marks the genomes where *F*_2_−*B*_2_ array is directed up, in (*PC*_2_,*PC*_3_) plane (directed down, respectively). In such capacity, the genomes with *CGC* triplet differ from those with *GCG* ones. Whether this difference is of a nature of things, or results from a bias of the database used in the study, should be examined further. One definitely could say there is no correlation between the pattern of orientation, triplet with the least standard deviation figure, and separation of plants on gymnosperm vs. angiosperm species.

**Table 2:** Distribution of orientation of patterns

	*CGC*	*GCG*
U	41	95
D	20	19

### Specific type of symmetry and coding regions

Consider now the number of points in clusters corresponding to the phases *F*_0_ through *B*_2_; obviously, they should be equal, or close, since the clusters differ in the reading frame shift of a triplet, only. Typical figures are the following: |*F*_0_|+|*B*_0_|=2489, |*F*_1_|+|*B*_1_|=2488, and |*F*_2_|+|*B*_2_|=2485 (here |·| means the capacity of a set, not an absolute value). The greatest standard deviation of the beam abundances is provided by *Hibiscus syriacus* (AC KP688069 in EMBL–bank), and the figure is 14.53. Reciprocally, the least figure (that is exactly zero) is provided by *Olimarabidopsis pumila* (AC AP009368).

**Fig. 10:**
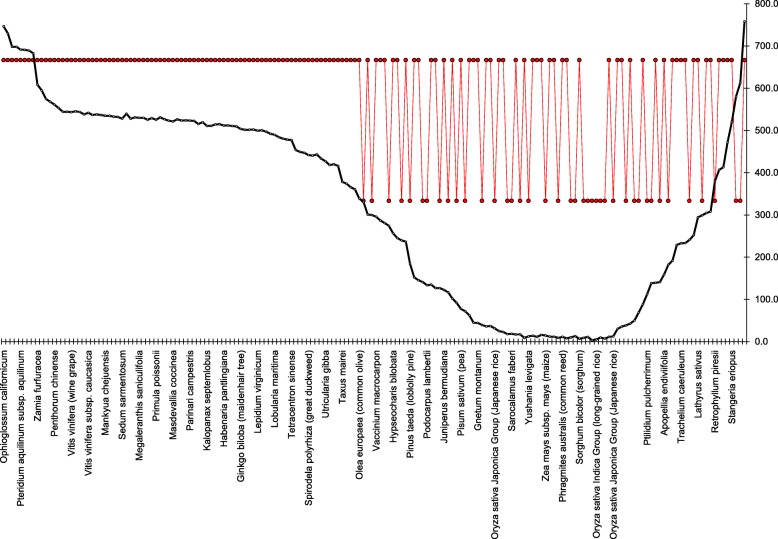
The bias within a phase, and the type of mirroring symmetry; see explanation in text

The difference between the phases |*F*_0_|−|*B*_0_|, |*F*_1_|−|*B*_1_| and |*F*_2_|−|*B*_2_| are of greater interest. These values vary from −1305 (averaged over three beams), for *Ophioglossum californicum* (AC KC117178) to 1387, for *Equisetum arvense* (AC GU191334). Figure 10 shows the relation of the bias in forward and backward coding regions occurrence, in different organisms, and the type of their mirroring symmetry. This figure shows the set of genomes ordered ascending on |*F*_0_|−|*B*_0_| figures; in other words, the left genome has |*F*_0_|−|*B*_0_|=−1362 (that is *Ophioglossum californicum*, AC KC117178), while the right one exhibits |*F*_0_|−|*B*_0_|=1382 (that is *Equisetum arvense*, AC GU191334). The solid black line in this figure shows the standard deviation of cluster abundances determined over all six phases; small red diamonds show the symmetry orientation: upper dots show U type, and lower ones show D type. It seems that the excess of the abundance of the fragments belonging to backward phases over those belonging to forward phases in 600 entities results in the unambiguous determination of U type symmetry orientation.

### What is beyond?

The study of statistical properties of DNA sequences still challenges researchers, and may brings many new findings. Here we have demonstrated basic structural difference of chloroplast genomes from the bacterial ones, manifested in the clustering in distribution of small formally identified fragments of a genome. Below are some issues that had fallen from the scope of this paper, while they are rather important to be considered in the nearest future.

#### Dark matter of a genome

Functional and evolution roles of the junk in a genome still is not clear in detail. It is extremely doubtful that junk has no matter in a genome, neither it plays a role in various and complicated biological processes involved into an inherited information processing and functioning. On the contrary, there are numerous evidences of high evolvement of non-coding (at least, protein non-coding) regions into regulatory of gene network [40–42], not speaking about the non-coding RNAs role. A short and simple paper [43] with remarkable title *Not junk after all* briefly enlists all the aspects of non-coding DNA regions in the life of a cell. Besides, the non-coding regions are a popular matter to develop some phylogenies [44–46] in various clades.

For some cases (see [36–38]), the removal of junk enforces the clustering of coding regions and makes easier the comprehension of the peculiarities standing behind. Yet, special efforts must be addressed to reveal the role and impact of junk regions of a genome on the processes mentioned above.

Not speaking about the differences in statistical properties of frequency dictionaries *W*_(3,3)_ (and *W*_(*m*,*n*)_, in general) observed for junk fragments of a genome vs. those observed for coding ones, one may expect the strong impact from the ratio of coding/non-coding parts occurred within a genome. For instance, here we report on mirror symmetry in mutual interlocation of six coding phases, for the frequency dictionary *W*_(3,3)_ developed for chloroplast genomes. Fig. 6 explicitly demonstrates an absence of such symmetry, for cyanobacteria genome, and this fact may result from a significant difference in the coding/non-coding ratio figures observed for these genetic systems.

#### Other chloroplast genomes

Here we present some results obtained on the careful examination of 178 genomes of ground plants. Yet, the generality of the observation awaits for further approval: first of all, one should study the chloroplast genomes of the organisms deviating rather far from the studied ones, in ecology (water plants, and algae, especially), physiology, taxonomy. Such systemic examination is the matter of the nearest future work of ours.

## Conclusion

Here we studied the structuredness of chloroplast genomes revealed trough the clustering of frequency dictionaries of considerably short fragments of a genome that were determined formally, with neither respect to the function encoded in a part of the genome fell into the fragment. The triplet dictionaries were developed, to cluster; these former counts triplets with no overlapping, while with no gaps between any two triplets. The fragments are distributed into eight distinct clusters: six of them gather the fragments falling into the coding regions, and differ in reading frame shift; the shift manifests in phase index of a fragment. The seventh cluster comprises the fragments falling into non-coding regions, and finally, the eighth cluster (so called *tail*) comprises the fragments with excessive *GC*-content value. These fragments correspond to the region where various tRNA and S RNA genes are concentrated; probably, this cluster includes also the “border” fragments (those that contain a border between coding and non-coding parts of a genome).

The clusters exhibit wonderful mirroring symmetry: the phase circuit in the forward and backward strands are counter-directed; this fact completely contradicts to the similar structure observed for bacteria, including cyanobacteria (which are stipulated to be the descendants of a common ancestor with chloroplasts). Such mirror symmetry yields a separation of the genomes into two groups: those with “up”-directed location of the cluster comprising *F*_2_ and *B*_2_ phases vs. those with “down”-directed; apparently, the threshold in the abundances of the phases gathered into a single cluster determines the direction of the *F*_2_−*B*_2_ cluster.

## Data Availability

Not applicable.
